# Disproportionate abdominal visceral fat mass reduction and complete reversal of cardiovascular remodelling accompany Roux-en-Y gastric bypass but not gastric banding - benefits beyond simply weight loss

**DOI:** 10.1186/1532-429X-18-S1-O135

**Published:** 2016-01-27

**Authors:** Jennifer J Rayner, Rajarshi Banerjee, Jane M Francis, Ravi V Shah, Venkatesh L Murthy, James Byrne, Stefan Neubauer, Oliver Rider

**Affiliations:** 1 grid.4991.50000000419368948University of Oxford, Oxford, UK; 2 grid.239395.70000000090118547Beth Israel Deaconess Medical Centre, Boston, MA USA; 3 University Hospital, Southampton, UK; 4 grid.214458.e0000000086837370University of Michigan, Ann Arbor, MI USA

## Background

It is emerging that distribution of body fat, and in particular visceral fat (VFAT) is more important in determining cardiovascular risk than total body fat percentage. Roux-en-Y gastric bypass (RYGB) may preferentially reduce visceral fat, thus providing a mechanism to study the impact of visceral fat on cardiovascular function. We sought to determine 1) whether visceral fat is related to adverse left ventricular (LV) remodelling and reduced aortic distensibility, and 2) whether RYGB, by preferentially targeting visceral fat, results in greater improvements in LV geometry and vascular function than gastric banding.

## Methods

159 subjects (body mass index (BMI) 18.5-59.2) without cardiovascular risk factors, underwent dual-energy X-Ray absorptiometry for fat distribution, MRI assessment (1.5T) for visceral fat and cardiac MR for LV geometry (mass and mass:volume ratio (LVMVR)). Aortic distensibility was assessed at 3 levels; the ascending and proximal descending aorta at pulmonary artery level and the abdominal aorta. 26 subjects underwent repeat testing 2.5 years following bariatric surgery (14 RYGB and 12 gastric banding).

## Results

After matching for BMI and total fat mass, patients were separated into two groups according to visceral fat. The high visceral fat group had greater concentric LV remodelling (LVMVR 0.93 ± 0.02 vs 0.77 ± 0.02; p < 0.001) and reduced aortic distensibility (by 18-27%; p < 0.01), suggesting that visceral fat rather than total fat mass is related to adverse cardiovascular remodelling.

**Figure 1:**
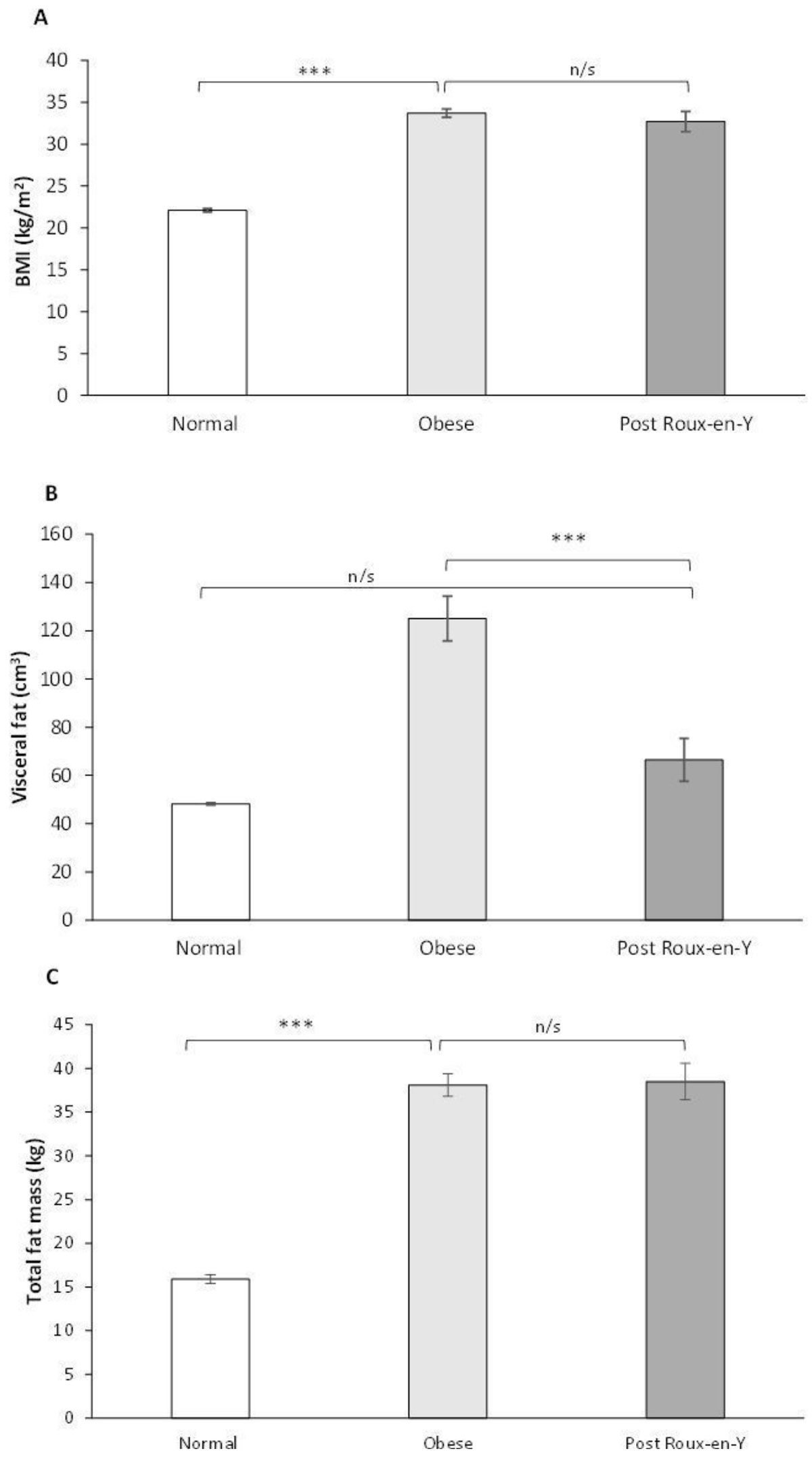
Despite BMI and total fat mass post RYGB remaining in the obese range (A and B), visceral fat is the same as individuals with normal BMI (C) (data represents mean values with SE bars; n/s indicates non-significant;*** indicates p < 0.001).

2.5 years after bariatric surgery, both groups had lost significant amounts of weight with BMI 32.7 ± 1.2 kg/m^2^ after RYGB and 32.6 ± 1.6 kg/m^2^ after gastric band (p = 0.94). Compared to gastric banding, RYGB resulted in greater reduction in visceral fat (62 ± 3% vs 31 ± 6% fall; p < 0.001, Figure 1) despite similar total fat mass loss (41 ± 3% vs 33 ± 5%; p = 0.92). Associated with visceral fat loss was greater regression of LV mass (23% vs 14% fall; p = 0.05) and greater improvement in aortic distensibility (95% vs 37%; p = 0.05). Importantly, following RYGB, visceral fat levels were both lower than in matched obese controls who had not undergone surgery (n = 36; VFAT post RYGB 66.5 ± 8.9 cm^2^ vs 125.2 ± 8.9 cm^2^; p < 0.05), and similar to normal weight controls (n = 73; VFAT 66.5 ± 8.9 cm^2^ post RYGB vs 48.2 ± 3.5 cm^2^; p > 0.05). Despite remaining obese post RYGB, LV mass and aortic distensibility were not different from normal weight controls (LV post RYGB 102.6 ± 5.0 g vs 103.1 ± 3.4 g; aortic distensibility post RYGB 8.54 ± 0.74 mmHg^-1^ vs 7.97 ± 0.33 mmHg^-1^; both p > 0.05).

## Conclusions

When matched for BMI and total fat mass, high visceral fat is related to concentric LV remodelling and aortic stiffness. RYGB is more effective than gastric banding at reducing visceral fat, reversing concentric LV remodelling and reducing aortic stiffness. This strongly suggests that targeted visceral fat reduction with RYGB should be considered to treat cardiovascular remodelling in obesity.

**Figure 2:**
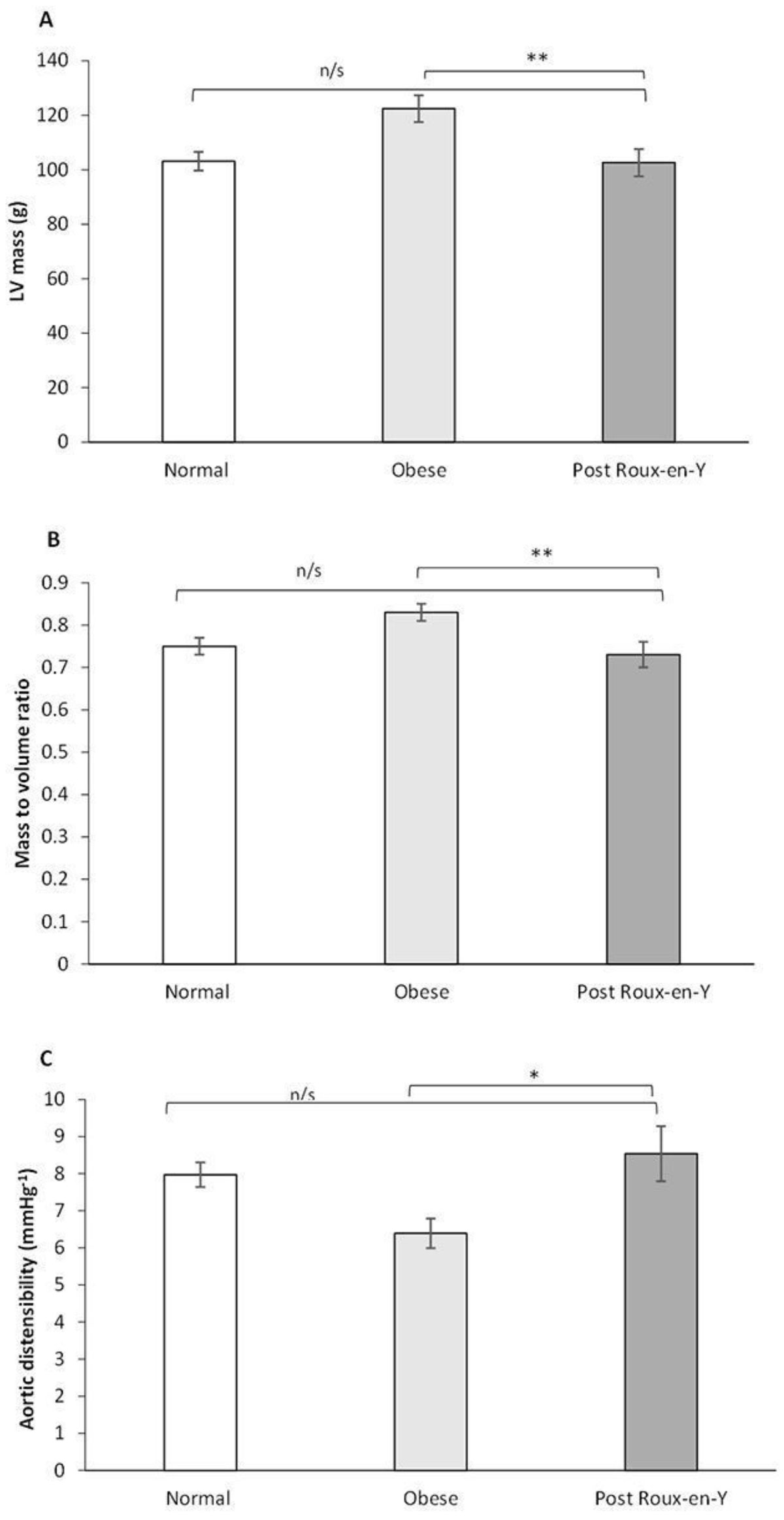
Left ventricular mass (A), and mass-to-volume ratio (B), as well as aortic distensibility in the descending aorta (C) return to values not significantly different from normal post RYGB (data represents mean values with SE bars; n/s indicates non-significant;** indicates p < 0.05; ** indicates p < 0.01; *** indicates p < 0.001).

